# Collective efficacy, alcohol outlet density, and young men’s alcohol use in rural South Africa

**DOI:** 10.1016/j.healthplace.2015.05.014

**Published:** 2015-07

**Authors:** Hannah H. Leslie, Jennifer Ahern, Audrey E. Pettifor, Rhian Twine, Kathleen Kahn, F. Xavier Gómez-Olivé, Sheri A. Lippman

**Affiliations:** a MPH–University of California, Berkeley, Division of Epidemiology, School of Public Health, Berkeley, CA, USA; b University of California, Berkeley, Division of Epidemiology, School of Public Health, Berkeley, CA, USA; c MPH–University of North Carolina at Chapel Hill, Department of Epidemiology, School of Public Health, Chapel Hill, North Carolina, USA and Medical Research Council/Wits University Rural Public Health and Health Transitions Research Unit (Agincourt); School of Public Health, Faculty of Health Sciences, University of the Witwatersrand Johannesburg, South Africa; d MPH-Medical Research Council/Wits University Rural Public Health and Health Transitions Research Unit (Agincourt); School of Public Health, Faculty of Health Sciences, University of the Witwatersrand Johannesburg, South Africa; e MPH, MBBCh-Medical Research Council/Wits University Rural Public Health and Health Transitions Research Unit (Agincourt); School of Public Health, Faculty of Health Sciences, University of the Witwatersrand Johannesburg, South Africa; f MBBCh, PhD, MSc-Medical Research Council/Wits University Rural Public Health and Health Transitions Research Unit (Agincourt); School of Public Health, Faculty of Health Sciences, University of the Witwatersrand Johannesburg, South Africa; g MPH-University of California, San Francisco, Center for AIDS Prevention Studies, Division of Prevention Science, Department of Medicine, San Francisco, CA, USA

**Keywords:** Alcohol, Alcohol outlet density, Collective efficacy, Rural South Africa, Agincourt health and demographic surveillance system

## Abstract

Alcohol use contributes to morbidity and mortality in developing countries by increasing the risk of trauma and disease, including alcohol dependence. Limited research addresses determinants of alcohol use beyond the individual level in sub-Saharan Africa. We test the association of community collective efficacy and alcohol outlet density with young men's drinking in a cross-sectional, locally representative survey conducted in rural northeast South Africa. Informal social control and cohesion show protective associations with men's heavy drinking, while alcohol outlet density is associated with more potential problem drinking. These findings provide initial support for intervening at the community level to promote alcohol reduction.

## Highlights

• We test social and structural factors as predictors of young men's alcohol use.
• Informal social control and cohesion are associated with reduced heavy drinking.
• Alcohol outlet density is associated with elevated potential problem drinking.
• Community factors may provide means to intervene on alcohol use and related harms.

## 1 Introduction

### 1.1 Alcohol use in South Africa

The harmful use of alcohol is a growing global public health priority. Alcohol consumption contributes to over 200 health conditions, including injury and both communicable and non-communicable diseases (World Health Organization, 2014a). Although the causal pathways are not fully elucidated, alcohol-related harms can be occasioned by the volume of alcohol consumed as well as through the particular pattern of drinking (Rehm et al., 2010). The broad effects of alcohol on risk of injury as well as communicable and non-communicable diseases are of particular salience in developing countries where other component causes of such outcomes are prevalent. Although levels of drinking tend to be lower in developing countries, the associated harms of alcohol use are disproportionately high (Room et al., 2002).

In South Africa, heavy alcohol consumption poses a serious risk to public health (Ferreira-Borges et al., 2015). Although over 40% of men in South Africa report abstinence from alcohol, consumption is high among drinkers; those who drink consume an average of over 30 l of pure alcohol (ethanol) per year (World Health Organization, 2014a), which is equivalent to nearly 3.5 U.S. pints of 5% alcohol-by-volume beer every day. This concentrated use results in considerable morbidity and mortality, particularly among men. As of 2012, an estimated 39,000 deaths were attributable to alcohol in South Africa (6.4% of all deaths), the vast majority of them among men (World Health Organization, 2014b). The contribution of alcohol use to alcohol use disorder (AUD), road traffic accidents, and liver cirrhosis alone accounted for approximately 5% of disability-adjusted life years (DALYs) among South African males in 2012 (World Health Organization, 2015). This represents only three of the health outcomes for which alcohol is a component cause and does not address morbidity and mortality from HIV, although increasing evidence of a role for alcohol in HIV transmission and progression to AIDS suggests that heavy alcohol consumption may be worsening South Africa's ongoing epidemic of HIV and AIDS (Hahn et al., 2011, Shuper et al., 2010, UNAIDS: Joint UN Program on HIV/AIDS, 2013, Woolf-King et al., 2013, World Health Organization, 2014a). Preventing alcohol-related harms and dependence is therefore a critical means of improving population health in South Africa.

### 1.2 Determinants of alcohol use

Alcohol use is a product of factors ranging from national historical context to individual genetic predisposition. Globally, level of alcohol consumption is associated with greater economic development between countries and higher socioeconomic status within countries (World Health Organization, 2014a). National and local policies on alcohol cost and availability as well as sanctions for alcohol-related offenses can shape individual consumption (Anderson et al., 2009). Individual-level characteristics consistently associated with alcohol use include age and gender; in South Africa as well as globally, alcohol consumption tends to increase with age and is much more common in men than women (Parry et al., 2005). Between national policy interventions and individual characteristics lie a number of potentially modifiable community factors, such as social norms around alcohol consumption, that may shape individual drinking. Although there is a long history of community-based prevention strategies in developed countries (Aguirre-Molina and Gorman, 1996), the relevance of this research to sub-Saharan Africa is only beginning to be assessed. Researchers recognize the need for prevention interventions that act on social and structural risk factors at the community level (Fritz et al., 2010, Kalichman, 2010). A more complete understanding of community causes of alcohol use in sub-Saharan Africa would facilitate effective population-level prevention of harmful alcohol use. We briefly review existing evidence, globally and in sub-Saharan Africa, of two potential community-level determinants of alcohol use: community collective efficacy and alcohol availability.

### 1.3 Collective efficacy and drinking

Motivated by theoretical work such as social disorganization theory, researchers have investigated links between community social context and drinking behavior (Bryden et al., 2013). Social disorganization theory posits that neighborhood structural conditions such as poverty and residential instability shape health outcomes through social factors like collective efficacy (Fulkerson et al., 2008). Collective efficacy is the capacity of a group to achieve a shared goal, and is comprised of two elements: working trust among community members (social cohesion) and, based on that trust, a mutual expectation to take action for shared interests (informal social control) (Sampson, 2003, Sampson et al., 1997). Although social factors have been linked to adolescent drinking, limited research addresses collective efficacy and adult alcohol use (Bryden et al., 2013). One study identified a protective association between informal social control and binge drinking among adults in Los Angeles, but found no association with cohesion (Carpiano, 2007). There is little research on this topic outside of the United States (Bryden et al., 2013).

In South Africa, initial examinations of social disorganization theory have produced mixed results. A small number of studies on the context of adolescent alcohol use support the relevance of community factors such as neighborhood dereliction in drinking behavior (Brook et al., 2011, Parry et al., 2004), with one study documenting a potential protective association between adolescents' perception of community affirmation and their consumption of home-brewed alcohol (Onya et al., 2012). Direct study of collective efficacy to date is scarce: a study in KwaZulu Natal employed a two-item measure of social cohesion that was correlated with lower social disorder (e.g., crime) and was associated with lower rates of adolescent sexual initiation, particularly for males (Burgard and Lee-Rife, 2009). However, social cohesion was weakly positively correlated with neighborhood disadvantage in this study, contrary to theoretical predictions. Cain et al. measured perceived collective efficacy among men and women in Cape Town as an individual's belief in their community's capacity to prevent HIV and found this to be associated with reduced frequency and quantity of alcohol use (Cain et al., 2013). It remains to be determined if community collective efficacy shapes alcohol use and HIV acquisition in South Africa.

### 1.4 Alcohol availability and drinking

Structural conditions such as alcohol availability comprise a second major focus of investigation into community-level determinants of alcohol use. Alcohol outlet density increases physical access to alcohol, which may lower alcohol prices and shape social behavior around drinking (Campbell et al., 2009). Ecologic studies from developed countries have shown overall alcohol consumption and alcohol-related harms to be higher in areas with greater outlet density (Popova et al., 2009). Findings have been mixed when assessing individual alcohol consumption, with studies in New Zealand and the United States finding no association between density of off-premise alcohol outlets (i.e. liquor stores) and average individual consumption (Connor et al., 2011, Pollack et al., 2005). A systematic review on availability of alcohol found the overall body of evidence to be inconclusive (Bryden et al., 2012). Nonetheless, the U.S. Guide to Community Preventive Services deems regulation of alcohol outlets a useful public health tool (Task Force on Community Preventive Services, 2009).

It is not yet known whether alcohol outlet density affects alcohol use in South Africa, where a majority of alcohol is sold at informal taverns or *shebeens*, as opposed to licensed on-premise (bar and restaurant) and off-premise alcohol outlets (Parry, 2010). A study from the Western Cape province found that socioeconomic deprivation is associated with a higher concentration of unlicensed outlets and fewer licensed outlets (Bowers et al., 2014), suggesting that, as in existing studies of outlet density, surrounding poverty may act as a confounder (Ahern et al., 2013, Connor et al., 2011, Pollack et al., 2005). Few studies address both social and physical environmental predictors of alcohol use within communities, and none to our knowledge has addressed these questions in South Africa.

### 1.5 Study aims

**Fig. 1:**
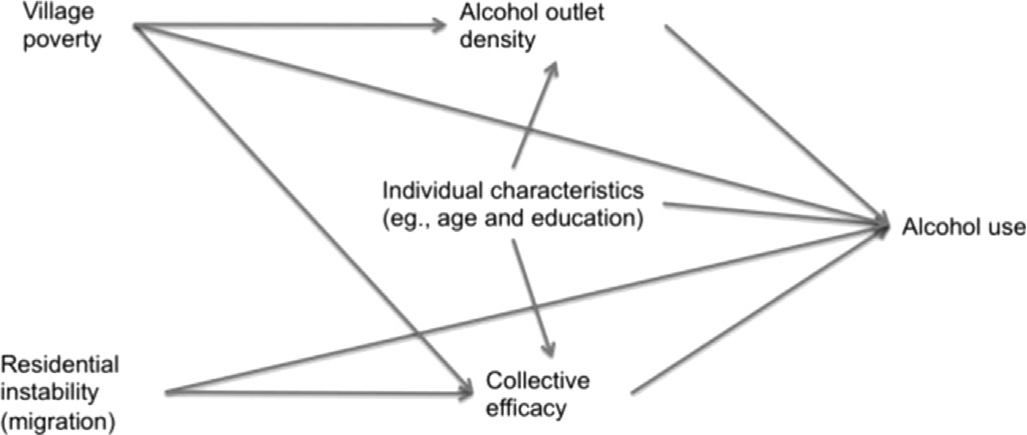
Causal framework of contextual factors affecting alcohol use.

We examine the relationship of community social and physical environmental factors with heavy alcohol consumption and potential problem drinking in a population-based sample of young men in rural South Africa. Heavy drinking is most consistently linked with alcohol-related morbidity and mortality, while the pattern and circumstances of drinking that comprise potential problem drinking are indicative of greater risk for future AUD. Fig. 1 shows the proposed causal model underlying this study. As posited by social disorganization theory, village structural determinants of poverty and instability can undermine collective efficacy, while lower collective efficacy may increase individual alcohol use. Similarly, village poverty may affect the location of alcohol outlets; outlet density in each village plausibly increases individual drinking. The probability that an individual lives in a given village and hence is exposed to the local alcohol outlet density and collective efficacy is a function of individual characteristics such as age and education, which also affect alcohol consumption. Other individual characteristics, such as psychosocial factors, are excluded from the model due to the assumption they do not affect individual selection into a village and hence are independent of exposure.

We test whether (1) collective efficacy and (2) alcohol outlet density affect individual heavy drinking and potential problem drinking. We hypothesize a protective association between collective efficacy and drinking outcomes, particularly potential problem drinking due to its inclusion of elements beyond the individual (e.g., expressions of concern about one's drinking). We hypothesize a harmful association between alcohol outlet density and drinking outcomes, especially heavy drinking since outlet density facilitates access to alcohol. This research can inform structural interventions at the community level, like those being implemented for HIV reduction in this region. If social factors such as collective efficacy do impact drinking behavior, interventions at the community level provide an optimal platform for addressing alcohol use. If alcohol availability plays a critical role in consumption patterns, policy interventions or community action should be targeted at limiting or better regulating alcohol outlets. The efforts undertaken by the South African government to confront alcohol-related harms at the national level (Parry et al., 2014, Parry, 2010) could be complemented by community-level approaches if modifiable factors associated with alcohol use are identified.

## 2 Materials and methods

### 2.1 Study site

The study is situated in the rural Agincourt sub-district of Mpumalanga province, South Africa, where the Medical Research Council / Wits University Rural Public Health and Health Transitions Research Unit (Agincourt) has been running a Health and Socio-demographic Surveillance System (HDSS) since 1992. The HDSS administers an annual census updating demographic and socio-economic data for all households in the area. At the time of this study, the area had approximately 90,000 people in 27 villages (Kahn et al., 2012). Mozambican immigrants comprise a sizable minority of the population, many of them from the Shangaan ethnic group predominant among native-born residents (Collinson, 2010). Unemployment is high, with only 29% of working age adults reporting employment in 2007 (Hunter et al., 2014). HIV prevalence peaks at over 45% for 35–39 year old adults (Gómez-Olivé et al., 2013).

### 2.2 Study procedures

This study combines Agincourt HDSS data with data from community and individual sources collected as part of a cluster randomized trial of an intervention called “One Man Can,” which aimed to reduce HIV risk among young men and women through community mobilization strategies (Lippman et al., 2013). In 2010, a community asset mapping exercise took place as part of formative research prior to initiation of the trial. Key informants convened in each village and identified current physical infrastructure throughout the village, including schools, clinics, sports fields, and alcohol outlets. This mapping resulted in a list of unique licensed and unlicensed alcohol-serving establishments within each village's boundaries. In 2012, a cross-sectional survey representative of all Agincourt HDSS members was conducted as the baseline for the trial. It consisted of a random sample of approximately 55 young adults (ages 18–35 years) per village from 22 of the sub-district villages, limited to one respondent per household. Individuals defined as temporary migrants, i.e. who had spent fewer than six months of the prior year as an area resident, were ineligible. Visits were made to 1826 households of a total of 2252 sampled for participation (81.1%); sample size was reached in some villages before the sample was exhausted. Sixty-nine percent (*n*=1,256) of households contacted included an eligible resident; 1181 of those eligible consented to enroll into the study (94.0%), 600 women and 581 men. Interviews were administered in English or Xitsonga (Shangaan) via computer-assisted personal interviews (CAPI) at the respondent's home. Sampling weights are applied to each response to account for probability of household selection and respondent selection within household.

The study was reviewed and approved by institutional review boards (IRBs) at the University of California, San Francisco; the University of North Carolina at Chapel Hill; and the University of the Witwatersrand, South Africa. The Mpumalanga Department of Health and Social Development Research Committee also approved the study. The analysis of de-identified data reported here was designated non-human subjects research by the IRB at the University of California, Berkeley.

### 2.3 Measures

Informal social control and social cohesion were measured on the baseline survey using items based on Sampson's collective efficacy scales (Sampson et al., 1997); items were added or adapted for local relevance, pilot tested, and revised to their final form (Table 1). Responses were coded from zero to two, with higher values representing increased likelihood on informal social control items and increased agreement with social cohesion items. Individual scores were calculated on each measure as the sum of standardized item responses; Cronbach's alpha was 0.88 for informal social control and 0.81 for social cohesion. Scores for respondents within each village were averaged to create continuous village-level metrics. We conducted sensitivity analyses using two alternative definitions of social exposures. First, to reduce the possibility of reverse causation due to drinkers perceiving village characteristics differently from others, village scores were recalculated excluding heavy drinkers and separately excluding potential problem drinkers. Second, to incorporate potential non-linear response patterns, individual scores for all respondents were created using the expected *a posteriori* (EAP) estimate from a generalized partial credit item-response model for each measure with sampling weights included (Masters and Wright, 1997). EAP reliability was 0.86 for informal social control and 0.78 for social cohesion in separate models (Wilson, 2005). Individual EAP scores were averaged within village.

Two types of alcohol outlets were identified during the community mapping: taverns (both licensed and *shebeens*) and bottle stores. Although bottle stores are nominally off-premise purveyors of alcohol, in these villages they often function as informal gathering places for alcohol consumption. As a result, we calculate outlet density as the total of both types of outlets divided by village area in square kilometers (km^2^).

Alcohol use was measured using the World Health Organization's Alcohol Use Disorders Identification Test (AUDIT), a well validated, 10-item screening tool for harmful and hazardous alcohol use that includes domains of alcohol consumption, symptoms of dependence, and related harms (Babor et al., 2001, Saunders et al., 1993). Each question is scored from zero to four points. Use of the AUDIT enabled us to capture both a metric of quantity of alcohol consumed and alcohol behaviors that indicate an individual is at risk of alcohol dependence. Heavy drinking was measured using the AUDIT-C, the subset of questions limited to frequency and amount of alcohol consumed, with a cut-point of four or more in accordance with past studies in South Africa and elsewhere (Bradley et al., 2007, Bush et al., 1998, Desmond et al., 2012, Peltzer et al., 2010). Individuals at risk of AUD were identified through a score of eight or above on the full AUDIT (Myer et al., 2008, Peltzer, 2006). Other alcohol variables included location where respondents typically drank, such as a tavern*,* restaurant, or home.

Additional covariates included age, education, marital status, being born outside of South Africa, and two metrics of individual poverty: earning no income in the past three months and experiencing food insecurity in the past 30 days. These two measures provide evidence of recent hardship, while educational attainment is an indicator of lifetime socioeconomic trajectory that does not necessarily predict recent employment; past studies have suggested that these measures are related to drinking outcomes in divergent ways (Parry et al., 2005). Agincourt HDSS census data were used to determine percent of village residents who were temporary migrants, defined as having spent under six months in the area over the previous year; percent employed, and percent of households with a female head. The percent of residents who were temporary migrants was used as a proxy for residential instability in this population. Percent employed and percent of female-headed household were used to capture village poverty level as female-headed households are more likely to be poor (Collinson, 2010).

### 2.4 Analysis

We assessed correlation of the collective efficacy sub-scales to determine whether they reflected a single underlying construct and correlation of collective efficacy with outlet density to determine whether the physical environment and social context were interrelated. Descriptive analysis included calculation of summary statistics of village characteristics and separate comparisons of characteristics of heavy-drinkers and potential problem drinkers respectively to all others using Chi square tests.

We examined the association of each exposure with heavy drinking and potential problem drinking as indicated in the causal framework. We adjusted the association between each collective efficacy sub-scale and drinking for village-level confounders suggested by social disorganization theory (poverty and residential instability); the associations of outlet density with alcohol outcomes controlled for village poverty. All analyses included individual characteristics likely to affect drinking and selection into a village: age, education, marital status, nationality, and poverty. We modeled age as years greater than 18 and as a quadratic term to best capture the non-linear relationship between age and each drinking outcome. Analyses employed logistic regression with sampling weights and robust clustered standard errors as well as a marginal modeling approach.

Marginal modeling enables estimation of an additive association that is interpretable in terms of population health (Ahern et al., 2009). We estimated the expected difference in prevalence of each drinking outcome for one standard deviation (SD) difference in each collective efficacy sub-scale by setting the exposure measures for all villages to one-half SD above and below the grand mean and using the regression model to predict outcomes under each setting to capture this contrast. We used the same procedure for outlet density, manipulating density to capture a difference of one outlet per square kilometer. Bias-corrected confidence intervals were generated from a clustered bootstrap with 10,000 resamples (DiCiccio and Efron, 1996). Regression and marginal modeling analyses were also run for sensitivity analyses incorporating the two alternative metrics for collective efficacy exposures described above.

We conducted one post-hoc analysis: we tested effect measure modification by computing the relative excess risk due to interaction (RERI) (VanderWeele and Knol, 2014). The RERI translates statistical interaction to the additive scale; a significant RERI may be indicative of causal interaction (Rothman et al., 2008). We consider *p*<0.20 statistically significant interaction. Analyses were conducted using the Test Analysis Module (TAM) package (Kiefer et al., 2015) in R 3.1.2 (R Foundation for Statistical Computing) for generating collective efficacy scores using item-response modeling and Stata 11.0 (StataCorp, College Station, TX, USA) for all other results.

## 3 Results

### 3.1 Village characteristics

Village size ranged from 0.72 km^2^ to 6.48 km^2^, with populations between 800 and 9000 at the time of the study. Taverns were more common than bottle stores, with up to six licensed outlets and *shebeens* per village compared to no more than two bottle stores. Villages contained an average of 1.37 alcohol outlets (range 0–3.24) per km^2^. Agincourt HDSS data affirmed the high level of poverty in this region, with an average of 41.6% (SD 3.2%) of households headed by a female and only 19.7% of adults employed (SD 1.77%). An average of 17.7% of residents were temporary migrants (SD 2.6%).

The 22 villages ranged from −0.28 to 0.36 on the standardized informal social control scale and from −0.39 to 0.38 on the standardized social cohesion scale. Though the two sub-scales are theorized as dimensions of collective efficacy, they were not strongly related in this context: correlation was 0.34 at the village level. Similarly, in the item-response model, a two-dimensional model showed no better fit than separate models. Alcohol outlet density was not correlated with either collective efficacy subscale. Each sub-scale was thus considered an independent village characteristic and analyzed individually.

### 3.2 Individual characteristics

Of the 581 men in the baseline sample, 343 (59.0%) reported any lifetime alcohol use (Table 2). Two hundred and one men (34.6%) were heavy drinkers and 140 (24.1%) were potential problem drinkers. The youngest men were least likely to be heavy drinkers; heavy drinking increased with higher educational attainment and with recent income, but was not significantly related to marital status. Just over half (110 of 201: 54.7%) of heavy drinking men were potential problem drinkers. As shown in Table 2B, similar traits distinguished potential problem drinkers: men under 20 were less likely to be potential problem drinkers, as were those who had never been married and those earning no income in the past three months.

### 3.3 Collective efficacy

As shown in Table 3, higher village informal social control was significantly associated with lower odds of heavy drinking after adjusting for both village-level confounders and individual covariates (*β*=−1.18, 95% confidence interval [CI] −2.26, −0.09). Village social cohesion similarly showed a significant association with heavy drinking: *β*=−1.07 (95% CI −1.82, −0.31). However, neither informal social control nor social cohesion was associated with potential problem drinking.

Table 4 displays the marginal modeling results for collective efficacy. A one SD higher level of community informal social control was associated with a −4.3% difference in prevalence of heavy drinking (95% CI −10.0, 0.7). One SD higher level of social cohesion was associated with a difference in prevalence of −4.2% (−9.6, −0.4) in heavy drinking among men across all villages. Sensitivity analyses were consistent with these findings: removing heavy drinkers from exposure assessment did not affect the magnitude or significance of the associations from the main analysis (results not shown); employing a more flexible measurement model slightly altered the estimated differences in heavy drinking associated with informal social control to −4.7% (−9.9, 0.3) and with social cohesion to −3.9% (−9.3, 0.2).

Marginal modeling results suggest non-significant differences of 1.3% (95% CI −6.5, 8.1) and 1.5% (−4.2, 8.3) in prevalence of potential problem drinking associated with one SD higher levels of informal social control and social cohesion respectively. These associations remained negligible and non-significant in both sensitivity analyses (not shown).

### 3.4 Alcohol outlet density

Results from multivariate regression of drinking outcomes on alcohol outlet density are shown in Table 5. Alcohol outlet density was not associated with prevalence of heavy drinking in either multivariate regression analyses (Table 5) or in marginal modeling (Table 6). However, higher outlet density was associated with increased risk of potential problem drinking. To better understand why outlet density would be unexpectedly associated with potential problem drinking but not heavy drinking, we assessed whether this risk differed between men who primarily drank at alcohol establishments and all other men, on the hypothesis that drinking behaviors and responses might differ based on the context of drinking. We found that drinking location acts as an effect measure modifier of the association between outlet density and probability of problem drinking (RERI for interaction of outlet density and primarily drinking at taverns=10.18, *p*=0.122) but not for heavy drinking (RERI=0.08, *p*=0.968). As a result, we present regression results for potential problem drinking stratified by drinking location in the right-hand panel of Table 5. Outlet density was positively associated with potential problem drinking only among the 281 men who drink in taverns (*β*=0.96, 95% CI 0.40, 1.52). Accounting for this interaction, the estimated prevalence of potential problem drinking was 27.6% under high alcohol outlet density and 18.4% under low density (Table 6). The marginal difference in potential problem drinking associated with a difference of one outlet per square kilometer in all villages is therefore 9.2% (95% CI 2.2%, 16.7%).

## 4 Discussion

This population-based study provides evidence that community social and physical environmental factors shape heavy alcohol consumption and potential problem drinking in South Africa. To our knowledge, no prior research has addressed the impact of both social and structural community characteristics on alcohol use within an adult population in South Africa. These results help to address this gap as well as the broader lack of research on potential community causes of alcohol use in low- and middle-income countries (Bryden et al., 2013, Bryden et al., 2012).

The association of collective efficacy measures with heavy drinking but not with potential problem drinking suggests that social disorganization theory may be relevant in explaining alcohol consumption in this context, albeit less relevant to dependence and harms. The finding that each measure of collective efficacy is associated with heavy drinking is unusual in research to date. Two studies in the United States and the Netherlands found some evidence of a protective association between moderate social cohesion and heavy drinking (Echeverría et al., 2008, Kuipers et al., 2012), while a study among adults in Los Angeles identified a protective association of informal social control against binge drinking, but no association of social cohesion (Carpiano, 2007). In contrast, studies of collective efficacy among adolescents in the United States (De Haan et al., 2010, Ennett et al., 2008, Maimon and Browning, 2012, Maimon and Browning, 2012) and of social cohesion among adults in New Zealand (Lin et al., 2012) have found no direct association with alcohol use. Differences in measurement of collective efficacy and alcohol use as well as varying analytic specifications make it difficult to compare results directly. The lack of association between collective efficacy measures and potential problem drinking in our study indicates that social factors offer at best a partial explanation of drinking behavior, and increase the possibility that the observed association with heavy drinking is erroneous. However, it is possible that the influence of the community social environment on young men is stronger in these rural villages, where employment opportunities are scarce compared to more urban, interconnected areas studied elsewhere. In addition, the community mapping exercise affirmed that village residents consider the village their community, ensuring that the units of analysis closely approximate individual perception of group identity and norms in the present study.

Further research on the application of social disorganization theory in this context is warranted. As part of the community mobilization intervention, study investigators have noted that the social cohesion measure fit their overall framework of mobilization, while informal social control was identified as a distinct construct (Lippman et al., 2013). The ongoing community mobilization intervention provides an opportunity to test whether cohesion and social control change together or separately. Post-intervention assessment will enable testing of any impact of such changes on alcohol use. Moreover, some elements of the original social disorganization theoretical framework do not operate identically in this setting. For example, regional patterns of migration and return are complex and have implications beyond residential turnover, such as the provision of remittances (Collinson et al., 2006, Collinson, 2010). The measure employed in this study may not capture the full range of influences of residential instability. As noted in other applications of social disorganization theory in South Africa, specific predictions based on theories developed in the United States may not hold true even if community factors do play a role in health behaviors (Burgard and Lee-Rife, 2009). Refinement of the conceptual framework tested here would strengthen future research in understanding the role of community factors.

Alcohol outlet density was associated with potential problem drinking but not with heavy drinking in this study; men who drank primarily in taverns were responsible for the observed association. These findings indicate that while individual levels of consumption may not be associated with increased availability of alcohol within villages, symptoms of dependence and alcohol-related harms may be. Similar results were obtained in a nationally representative study in New Zealand, where outlet density was related to alcohol-related harms without being associated with average consumption (Connor et al., 2011). One potential explanation for the lack of association between village outlet density and consumption is regional alcohol availability outside of taverns and liquor stores: one major site of drinking is weekly *muchongolo* (traditional) dance events, which rotate throughout the villages and at which residents of many villages congregate. Home-brewed alcohol as well as alcohol provided by informal vendors is available at the dances (Niehaus and Stadler, 2004), providing a source of consumption independent of outlet density in one's home village.

The association of outlet density with potential problem drinking suggests that formal and informal taverns may shape social behavior around drinking in ways that result in greater perceived dependence and harms. A difference of just one outlet per square kilometer was associated with a meaningful difference in prevalence of potential problem drinking. This evidence bolsters the existing focus on *shebeens* in South Africa as critical sites of individual risk and of potential intervention (Cain et al., 2012, Morojele et al., 2006, Scott-Sheldon et al., 2014). Additional metrics of drinking behavior and direct measurement of alcohol-related harms would provide greater insight on these relationships.

Several limitations should be considered in interpreting study findings. The measured confounders are unlikely to represent all shared antecedents of exposures and individual drinking. Results would be biased if outlet density or collective efficacy in one village affected drinking behavior in other villages in the study; such contamination is more plausible for outlet density than collective efficacy, as an individual could choose to travel to a village with greater availability of alcohol. Although outlet density was measured prior to the individual survey, collective efficacy was measured simultaneously with drinking. The observed association could therefore be due to reverse causation, with drinking behavior eroding collective efficacy. The sensitivity analysis excluding heavy drinkers from the calculation of the collective efficacy measure does account for how their perceptions of the village could impact results, but not for any effect heavy drinkers have on neighbors' perceptions or for their relocation to less cohesive villages.

Measurement error could bias the findings in a number of ways. Village collective efficacy is based on perceptions only of those aged 18–35 years. This age group was selected because the parent intervention study focuses on changing the social environment shaping sexual health for young women and their partners; however, it may result in incomplete measurement of village characteristics. The AUDIT is a screening tool and hence imperfectly sensitive and specific (Bush et al., 1998). Self-report of alcohol use may be affected by social desirability bias and by uncertainty around standard drink size when consumption occurs in less formal settings. Site-specific research into drink size and patterns of consumption would strengthen future research (Nayak et al., 2008). However, there is little reason to believe that responses to the AUDIT differ systematically by village factors, decreasing the chance of misclassification biasing the estimates unpredictably.

This study builds on several design and analysis strengths to provide new insight into the community context of drinking behavior. The results presented draw on a representative sample from a population-based sampling frame, rendering the findings more generalizable than data from studies using clinic-based populations or convenience samples. Alcohol outlet density was assessed through community mapping in order to capture a full picture of drinking establishments, both licensed and unlicensed, in this area. Moreover, measures of collective efficacy employed were grounded in the theoretical work undertaken in the United States and adapted to this context to provide comprehensive, reliable metrics for use at the community level (Lippman et al., 2013). Sensitivity analyses of potential reverse causality and exposure misclassification were consistent with the main analysis. Marginal modeling enabled calculation of population estimates of the difference in drinking corresponding to changes in exposure that could public health interventions could plausibly effect, such as the one conducted at the site. Estimates of potential change can help to guide choice of intervention components.

The findings presented here provide the first evidence of associations between community social and physical environmental factors and young men's alcohol use in South Africa. They suggest that community social factors such as cohesion and perhaps informal social control are related to men's heavy drinking. Moreover, the results suggest that a modest difference in density of drinking establishments is associated with a substantial amount of potential problem drinking. Identifying upstream factors that could mitigate alcohol-related harms and dependence opens new opportunities to improve population health in South Africa.

## Contributors

AP and SL designed the research protocol. KK, XGO and RT were responsible for research implementation. SL designed social environmental measures; JA oversaw the analytic approach. HL conceptualized the research question, analyzed the data and drafted the article. All of the authors provided input on and edited the article.

## Competing interests

The authors have declared that no competing interests exist.

## Figures and Tables

**Table 1:** Items used to measure collective efficacy.

Informal social control: *Would you say it is very likely, somewhat likely, or unlikely that your neighbors could be counted on to intervene in various ways if:*
Children were skipping school and hanging out on a street corner?
Children were breaking windows on a local building/destroying public property?
Children were showing disrespect to an adult?
A fight broke out at the pension point?
The local school closed down the feeding scheme?
A family didn't have enough food?
The neighborhood water tank was broken?
An elderly person was robbed?
Social cohesion: *For each of the following statements, please tell us if you agree a lot, somewhat agree, or do not agree at all with the statement:*
People in this village are willing to help their neighbors.
This is a close-knit village.
People in this village can be trusted.
People in this village generally get along well with each other.
People in this village share the same values.
People in this village look out for each other.

**Table 2:** Characteristics of young men by current drinking status (*N*=581).

A. Heavy drinkers compared to non-drinkers and light drinkers			B. Potential problem drinkers compared to non-drinkers and non-problem drinkers		
	Non-drinker or light drinker (*n*=380) N (col %)	Heavy drinker (*n*=201) N (col %)		Non-drinker or non-problem drinker (*n*=441) N (col %)	Potential problem drinker (*n*=140) N (col %)

Age⁎⁎			Age⁎⁎		
18–20	193 (50.8)	69 (34.3)	18–20	218 (49.4)	44 (31.4)
21–25	109 (28.7)	74 (36.8)	21–25	132 (29.9)	51 (36.4)
26–30	45 (11.8)	39 (19.4)	26–30	54 (12.2)	30 (21.4)
31–35	33 (8.7)	19 (9.5)	31–35	37 (8.4)	15 (10.7)
					
Education⁎⁎			Education⁎		
Primary or less	45 (11.8)	18 (9.0)	Primary or less	45 (10.2)	18 (12.9)
Some secondary	239 (62.9)	108 (53.7)	Some secondary	276 (62.6)	71 (50.7)
Completed secondary or above	96 (25.3)	75 (37.3)	Completed secondary or above	120 (27.2)	51 (36.4)
					
Marital status			Marital status⁎⁎		
Never married	324 (85.3)	163 (81.1)	Never married	378 (85.7)	109 (77.9)
Married (legal or traditional)	41 (10.8)	23 (11.4)	Married (legal or traditional)	47 (10.7)	17 (12.1)
Separated, divorced or widowed	15 (4.0)	15 (7.5)	Separated, divorced or widowed	16 (3.6)	14 (10.0)
Born outside South Africa	43 (11.3)	18 (9.0)	Born outside South Africa	51 (11.6)	10 (7.1)
Earned no income within three months⁎	278 (73.2)	128 (63.7)	Earned no income within three months⁎⁎	326 (73.9)	80 (57.1)
Experienced food insecurity within 30 days	11 (2.9)	8 (4.0)	Experienced food insecurity within 30 days	13 (3.0)	6 (4.3)
Potential problem drinker⁎⁎	30 (7.9)	110 (54.7)			

*Note:* Chi square test *p* values

⁎⁎ *p*<0.01.

⁎ *p*<0.05.

**Table 3:** Multivariate logistic models of the relationship between collective efficacy measures and alcohol use among men (*N*=581).

	Heavy drinking		Potential problem drinking	
	Informal social control model	Social cohesion model	Informal social control model	Social cohesion model
	Coeff. (95% CI)	Coeff. (95% CI)	Coeff. (95% CI)	Coeff. (95% CI)
Informal social control	−1.18 (−2.26, -0.09)	–	0.43 (−1.42, 2.28)	–
Social cohesion	–	−1.07 (−1.82, −0.31)	–	0.46 (−1.00, 1.92)
Age (years over 18)	0.18 (0.01, 0.36)	0.18 (0.00, 0.36)	0.16 (0.01, 0.31)	0.16 (0.01, 0.31)
Age squared	−0.01 (−0.02, 0.00)	−0.01 (−0.02, 0.00)	−0.01 (−0.02, 0.00)	−0.01 (−0.02, 0.00)
				
Education				
Primary or less	REF	REF	REF	REF
Some secondary	−0.26 (−1.10, 0.59)	−0.25 (−1.07, 0.58)	−1.02 (−2.24, 0.20)	−1.02 (−2.26, 0.21)
Completed secondary or above	0.26 (−0.58, 1.10)	0.30 (−0.52, 1.12)	−0.42 (−1.49, 0.65)	−0.44 (−1.50, 0.62)
				
Marital status				
Never married	REF	REF	REF	REF
Married (legal or traditional)	0.24 (−0.55, 1.02)	0.19, (−0.61, 0.99)	−0.24 (−1.26, 0.78)	−0.22 (−1.29, 0.85)
Separated, divorced, widowed	−0.06 (−1.05, 0.93)	0.00 (−1.00, 1.00)	0.67 (−1.05, 2.40)	0.65 (−1.10, 2.41)
Born outside South Africa	−0.01 (−0.84, 0.82)	0.09 (−0.70, 0.89)	−0.54 (−1.75, 0.68)	−0.58 (−1.78, 0.62)
Earned no income within 3 months	−0.36 (−0.77, 0.05)	−0.32 (−0.73, 0.08)	−0.19 (−0.76, 0.37)	−0.20 (−0.75, 0.35)
Experienced food insecurity within 30 days	0.64 (−0.69, 1.98)	0.56 (−0.80, 1.92)	0.16 (−0.93, 1.24)	0.18 (−0.92, 1.28)
Village % female-headed households	0.03 (−0.06, 0.11)	−0.02 (−0.08, 0.05)	0.06 (−0.06, 0.18)	0.08 (−0.02, 0.18)
Village % employed	−0.05 (−0.25, 0.16)	−0.09 (−0.26, 0.08)	−0.02 (−0.29, 0.25)	0.00 (−0.21, 0.21)
Village % migrant	0.12 (−0.04, 0.27)	0.17 (0.07, 0.27)	0.01 (−0.21, 0.23)	−0.01 (−0.16, 0.14)
Intercept	−3.25 (−6.59, 0.09)	−1.48 (−3.85, 0.90)	−3.22 (−7.72, 1.29)	−3.87 (−7.32, −0.41)

**Table 4:** Predicted population prevalence of alcohol use by level of collective efficacy.

Exposure:	Heavy drinking		Potential problem drinking	
	Informal social control	Social cohesion	Informal social control	Social cohesion
High (0.5 SD above mean)	30.2%	29.8%	23.8%	24.1%
Mean	32.3%	31.9%	23.1%	23.3%
Low (0.5 SD below mean)	34.5%	34.0%	22.5%	22.6%
Difference (95% CI)	−4.3% (−10.0, 0.7)	−4.2% (−9.6, −0.4)	1.3% (−6.5, 8.1)	1.5% (−4.2, 8.3)

**Table 5:** Multivariate logistic models of the relationship between alcohol outlet density and alcohol use (*N*=581).

	Heavy drinking	Potential problem drinking	
		Tavern drinkers (*N*=281)	Non-tavern drinkers (*N*=292)
	Coeff. (95% CI)	Coeff. (95% CI)	Coeff. (95% CI)
Alcohol outlet density	−0.13 (−0.64, 0.39)	0.96 (0.40, 1.52)	−0.36 (−1.19, 0.46)
Age (years over 18)	0.18 (0.02, 0.35)	0.04 (−0.13, 0.20)	0.25 (−0.10, 0.59)
Age squared	−0.01 (−0.02, 0.00)	0.00 (−0.01, 0.01)	−0.01 (−0.03, 0.02)
			
Education			
Primary or less	REF	REF	REF
Some secondary	−0.03 (−0.84, 0.78)	−1.09 (−.42, 0.23)	−1.17 (−2.96, 0.61)
Completed secondary or above	0.46 (−0.35, 1.27)	−0.36 (−1.32, 0.61)	−1.01 (−2.91, 0.88)
			
Marital status			
Never married	REF	REF	REF
Married (legal or traditional)	0.10 (−0.72, 0.92)	0.14 (−1.45, 1.73)	−2.51 (−5.57, 0.55)
Separated, divorced, widowed	−0.16 (−1.15, 0.83)	0.86 (−0.91, 2.63)	−1.23 (−4.68, 2.23)
Born outside South Africa	0.07 (−0.76, 0.90)	−0.85 (−1.72, 0.02)	−1.25 (−3.87, 1.38)
Earned no income within 3 months	−0.23 (−0.63, 0.17)	−0.16 (−0.92, 0.59)	−0.10 (−1.71, 1.51)
Experienced food insecurity within 30 days	0.64 (−0.65, 1.93)	0.52 (−1.25, 2.30)	a
Village % female-headed households	0.03 (−0.08, 0.14)	0.19 (0.05, 0.34)	0.01 (−0.13, 0.15)
Village % employed	0.01 (−0.16, 0.19)	−0.12 (−0.31, 0.07)	0.03 (−0.18, 0.25)
Intercept	−2.53 (−6.76, 1.69)	−6.65 (-13.52, 0.21)	−3.09 (−8.49, 2.31)

a Variable omitted due to collinearity with outcome

**Table 6:** Predicted population prevalence of alcohol use by alcohol outlet density.

	Heavy drinking	Potential problem drinking
High (0.5 outlets/km^2^ above mean)	30.1%	27.6%
Mean	31.4%	22.8%
Low (0.5 outlets/km^2^ below mean)	32.7%	18.4%
Difference (95% CI)	−2.6% (−12.6, 10.2)	9.2% (2.2, 16.7)
